# Depression in Working Adults: Comparing the Costs and Health Outcomes of Working When Ill

**DOI:** 10.1371/journal.pone.0105430

**Published:** 2014-09-02

**Authors:** Fiona Cocker, Jan M. Nicholson, Nicholas Graves, Brian Oldenburg, Andrew J. Palmer, Angela Martin, Jenn Scott, Alison Venn, Kristy Sanderson

**Affiliations:** 1 School of Population and Global Health, University of Melbourne, Melbourne, Australia; 2 Judith Lumley Centre, La Trobe University, Melbourne, Australia; 3 School of Public Health, Queensland University of Technology, Queensland, Australia; 4 Menzies Research Institute Tasmania, University of Tasmania, Hobart, Australia; 5 Tasmanian School of Business and Economics, University of Tasmania, Hobart, Australia; 6 School of Psychology, University of Tasmania, Hobart, Tasmania, Australia; institute of Health Policy and Management, Netherlands

## Abstract

**Objective:**

Working through a depressive illness can improve mental health but also carries risks and costs from reduced concentration, fatigue, and poor on-the-job performance. However, evidence-based recommendations for managing work attendance decisions, which benefit individuals and employers, are lacking. Therefore, this study has compared the costs and health outcomes of short-term absenteeism versus working while ill (“presenteeism”) amongst employed Australians reporting lifetime major depression.

**Methods:**

Cohort simulation using state-transition Markov models simulated movement of a hypothetical cohort of workers, reporting lifetime major depression, between health states over one- and five-years according to probabilities derived from a quality epidemiological data source and existing clinical literature. Model outcomes were health service and employment-related costs, and quality-adjusted-life-years (QALYs), captured for absenteeism relative to presenteeism, and stratified by occupation (blue versus white-collar).

**Results:**

Per employee with depression, absenteeism produced higher mean costs than presenteeism over one- and five-years ($42,573/5-years for absenteeism, $37,791/5-years for presenteeism). However, overlapping confidence intervals rendered differences non-significant. Employment-related costs (lost productive time, job turnover), and antidepressant medication and service use costs of absenteeism and presenteeism were significantly higher for white-collar workers. Health outcomes differed for absenteeism versus presenteeism amongst white-collar workers only.

**Conclusions:**

Costs and health outcomes for absenteeism and presenteeism were not significantly different; service use costs excepted. Significant variation by occupation type was identified. These findings provide the first occupation-specific cost evidence which can be used by clinicians, employees, and employers to review their management of depression-related work attendance, and may suggest encouraging employees to continue working is warranted.

## Introduction

The economic cost of depression is largely due to related work impairment, disability [1], and lost productivity from sickness absenteeism and presenteeism (continuing to work when ill) [2]. Presenteeism is common [3], more costly than absenteeism [3], [4], and may account for up to 80% of depression-related lost productive time [2]. Internationally, this equates to an estimated 35.7 billion USD [2], 15.1 billion UK pounds [5] and 12.6 billion Australian dollars [6] annually. However, the health outcomes of presenteeism are less established [7]. Although presenteeism has been shown to increase the incidence of serious coronary events [8], and predict poor self-rated health [9] and future sickness absence [10], it may also confer health benefits via supervisor or colleague support and a maintained daily routine [11]. In fact, its potential health benefits may outweigh any negative health outcomes and economic costs.

Evidence regarding the economic costs and health benefits of absenteeism *and* presenteeism is essential to inform the design of workplace depression management strategies [12], particularly those focused on promotion and prevention. At present, the right balance between absenteeism and presenteeism for employees with depression is unknown. Therefore, current clinical practice guidelines for employers or employees seeking informed advice about when continued work attendance is optimal are lacking. Further, despite awareness that work characteristics and demands can influence employee attitudes to work attendance or render them either unable or reluctant to take time off when sick [13], evidence of whether the costs and health consequences of absenteeism and presenteeism differ by occupation is scarce. Therefore, it is unclear whether work attendance recommendations should be tailored to different job types.

This study used population level data and a Markov cohort simulation approach to compare the costs and health outcomes of working while ill versus work absence over time amongst employed Australians reporting lifetime major depression. The model was amended to quantify variations across occupation (blue vs. white collar). With the information provided this study aimed to: i) determine whether continuing to work when ill or taking a planned, short-term sickness absence is the more cost-effective decision for employees reporting depression; and ii) determine whether subsequent recommendations should be altered by occupation type.

## Method

### Definition of scenarios and type of Analysis

We conducted an epidemiologic-based, analytic modelling study, using cohort simulation and a state-transition Markov model, to compare the costs and health outcomes of working while experiencing depression versus taking a sickness absence. The decision analysis approach used in this study requires presenteeism and absenteeism to be defined as mutually exclusive scenarios. Presenteeism, was therefore defined as the absence of absenteeism, consistent with previous approaches [8]. In other words, no reported depression-specific, work- and role-functioning disability days in response to the NSMHWB depression module item “About how many days out of 365 in the past 12 months were you totally unable to work or carry out your normal activities because of your (sadness/or/discouragement/or/lack of interest)?”. Absenteeism, was the converse. Therefore, this analysis is based on two assumptions; a) all employed individuals with 12-month depression will experience impairment relevant to their work; and b) the categories of 12-month absenteeism and presenteeism are mutually exclusive. This method was selected as it provides a measure of depression-specific disability days and therefore removes the possible influence of co-morbid disorders of work attendance decisions.

Two subsequent models determined whether outcomes differed for blue- versus white-collar workers. All models were identically structured and generated using Data TreeAge Pro software (Williamstown, Mass.). Cohort simulation was deemed appropriate as it synthesises best available evidence to address difficult-to-answer questions, and is ideal when experimental trials are not ethical or feasible. Cohort simulation is commonly used in health economics, and related clinical and epidemiological research, to model future costs and outcomes of patients, groups or populations under alternative scenarios such as different treatment options [25] and is unique in that it is able to predict cross-sectional data and simulate life courses of people, providing longitudinal outcomes. A wide range of evidence is usually included, such as epidemiologic surveys, meta-analyses, and high-quality single studies in order to determine the benefits and costs beyond time horizon of existing data [26] [37].

### Analytic Structure and Time Horizon

**Figure 1:**
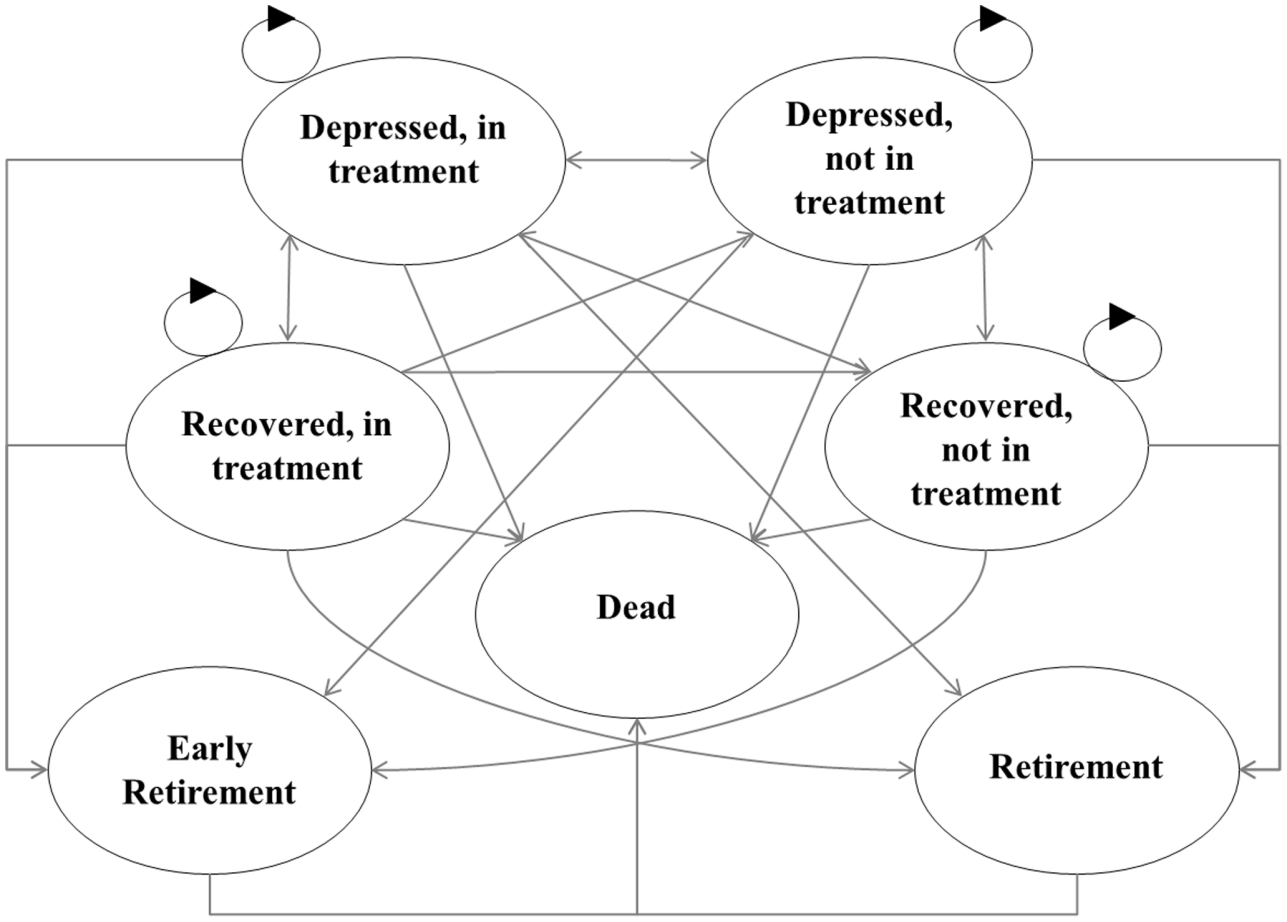
State transition Markov model diagram.

A hypothetical cohort of employees (N = 1000) occupied and moved between seven health states over time according to probabilities (Figure 1) [14]. A 3-month cycle length was chosen to reflect the natural history of depression, and the selected health states are clinically relevant and informed by related research [14]. Where relevant, health states were assigned lost productive time, job turnover, and health service use costs, and a utility value consistent with a depression diagnosis (depressed/not depressed) and treatment status (in treatment/not in treatment). The number of people and the amount of time they spent in each health state determined the aggregate costs and health outcomes at the conclusion of the model. Costs and health outcomes were considered from the societal perspective over 1-year, and extended to a 5-year time horizon to produce results relevant to employers' decision-making time frames i.e. those interested in improving outcomes for their current employees.

### Data Sources

Probabilities and costs were derived from our primary epidemiological data source, the National Survey of Mental Health and Wellbeing (2007) (NSMHWB) [15], or published literature. The NSMHWB is a stratified, random household survey conducted by the Australian Bureau of Statistics (ABS) to determine lifetime and 12-month prevalence estimates of affective and other common psychiatric disorders within the Australian population. To estimate the prevalence of specific mental disorders, the NSMHWB used the World Mental Health Survey Initiative version of the World Health Organization's Composite International Diagnostic Interview, version 3.0 (WMH-CIDI 3.0). This is a comprehensive interview used to assess the lifetime, 12-month, and 30-day prevalence of selected mental disorders by measuring symptoms and their impact on day-to-day activities. It provides an assessment of mental disorders based on the definitions and criteria of two classification systems: the Diagnostic and Statistical Manual of Mental Disorders, Fourth Edition (DSM-IV); and the WHO International Classification of Diseases, Tenth Revision (ICD-10). Both the DSM-IV and the ICD-10 have sets of criteria, necessary for diagnosis, which specify the nature and number of symptoms required, the level of distress or impairment required, and the exclusion of cases where symptoms can be attributed to general medical conditions, such as a physical injury, or to substances, including alcohol. The WMH-CIDI 3.0 was also used to collect information on the course, onset, recency and persistence of symptoms of mental disorders, the impact of mental disorders on home, work, relationship and social functioning, and treatment seeking and access to adequate treatment.

The NSMHWB received a response rate of 60% (N = 8841) and data were weighted to represent the projected Australian adult population (N = 16 015 300) thus ensuring the data and findings derived from this survey are generalisable to the total in scope population. More specifically, weighting adjusts results from a sample survey to infer results for the total in-scope population by allocating a ‘weight’ to each sample unit corresponding to the level at which population statistics are produced, e.g. household or person level. This weight is considered an indication of how many population units are represented by the sample unit. ABS household surveys are calibrated to population benchmarks by state, part of state, age and sex. Initial person weights were simultaneously calibrated to population benchmarks for state by part of state, age, sex, state by household composition, state by educational attainment, and state by labour force status. Household weights were derived by separately calibrating initial household selection weights to the projected household composition population counts of households containing persons aged 16–85 years, who were living in private dwellings in each state and territory of Australia, at 31 October 2007.

Occupation type was derived from the employment component of the NSMHWB, which was summarised according to the Australian and New Zealand Standard Classification of Occupation (ANZSCO) [16]. Data from published studies determined the probability [17] and cost [18] of depression-related job turnover, mean presenteeism days [19], and absenteeism and presenteeism-related lost productive time costs [2], [20].

### Initial Probabilities

The NSMHWB provided the major depression diagnosis used to distribute the cohort among health states (Figure 1), and the reported depression-specific disability days used to determine distribution between absenteeism and presenteeism scenarios. The probability of being in each of the health states for a hypothetical white or blue collar worker reporting absenteeism are presented in Table S2, initial probabilities for white and blue collar workers reporting presenteeism are presented in Table S3. (see Tables S1, S2, S3) [21]. Models only included individuals with depression, as determined by current 12-month symptoms or any lifetime experience. Individuals were defined as ‘depressed’ if they reported 12-month depression symptoms, or ‘recovered’ if they reported lifetime depression without 12-month symptoms. ‘In treatment’ referred to self-reported contact with a health professional for a mental health problem any time in the last 12-months [15]. Individuals started the simulation process in a ‘depressed’ or a ‘recovered’ state (Figure 1).

### Transition Probabilities

Transition probabilities were derived from relevant secondary sources (see Table S1, S2, S3), applied in each successive 3-month cycle, and governed the cohort's movement between health states over time (see Table S1, S2, 3). Tables S1, S2 and S3 demonstrate how they differed according to occupation type (Table S1, S2, S3). Remission (with and without treatment) determined movement from a ‘depressed’ to a ‘recovered’ state, and relapse (with and without treatment) was the converse. The transition probabilities were derived from published findings from a national survey of the US population [2], [4], (see Table S1) in which diagnoses were made using the Primary Care Evaluation of Mental Disorders (PRIME-MD) instrument. The PRIME-MD has been shown to have excellent agreement with clinical diagnoses made using the lengthier diagnostic interviews such as the CIDI [35], [36]. Treatment initiation probabilities determined movement from a ‘not in treatment’ to an ‘in treatment’ state. Age- and sex-specific mortality/survival rates determined movement to the ‘deceased’ state. All depression states had an increased mortality rate due to risk of suicide, and an increased risk of early retirement (before the age of 50). The deceased and retirement health states were absorbing states, which individuals could not leave once entered.

### Costs

Lost productive time, job turnover, depression-related service use and antidepressant medication costs were assigned to each health state (see Table S1, S2, S3). They were based on the probability of various cost-incurring events being experienced, the number of times that event occurred, and the unit cost assigned to that event. All costs were in 2007 Australian dollars (AUD), to reflect the reference year of the NSMHWB. Lost productive time costs involved multiplying the number of depression-specific absenteeism and presenteeism days, adjusted to a 3-month estimate, by the average daily wage. Daily wage, weekly wage and annual salary were calculated using the Australian Bureau of Statistics estimates of employee earnings and hours averaged across all occupations, and blue collar and white collar occupations separately, as defined by ANZSCO [16]. Depression-related job turnover costs included the recruitment, hiring and training costs of replacing an employee who is terminated or voluntarily leaves. The job turnover probability estimate, although deemed the best available [22], was from a sample considered unrepresentative of the general population. Therefore, probabilities were restricted to ‘depressed in treatment’ states [17]. The NSMHWB provided depression-specific service use and antidepressant medication costs. Number of contacts in the past year with general practitioners, psychologists, psychiatrists, mental health nurses and alternative therapists were costed using Australia's national health insurance scheme information (the Medicare Benefits Schedule). Reported 2-week antidepressant medication use was converted to 3-month probability estimates. As the type/s of antidepressant used, and duration and dosage were unknown, prescriptions were costed for 3-months using the medication type (Selective Serotonin Reuptake Inhibitors) and dosage recommended under optimal care [23].

### Health Outcomes

Quality-adjusted life years (QALYs), a combination of quality (measured using utilities) and duration of life, were the primary measure of health outcome (see Table S1, S2, S3). Utilities are a global measure of the value attached to each health state and ideal to capture the broad effects on health and wellbeing possible from presenteeism and absenteeism [7], [8]. The applicability of utility-weighted, population health outcomes to depression has been demonstrated [24]. The NSMHWB [15] provided utilities derived from the Assessment of Quality of Life-4D (AQoL-4D) [25], a validated measure, able to detect subtle quality-of-life differences in areas including mental health [26].

### Sensitivity Analysis

Probabilistic sensitivity analysis using Monte Carlo simulation determined the total costs and health outcomes of all models. Values for all model parameters were sampled from specified distributions. These were beta distributions for probabilities, gamma distributions for costs and uniform distributions when true functional form was unknown as recommended by published guidelines for decision modelling in health economic evaluation [27]. Expected costs and health outcomes were calculated for a hypothetical cohort of 1000 workers. Re-sampling from each distribution and recalculating the costs and health outcomes from the model generated a distribution of the estimated values. 95% credible intervals were estimated from the simulated data. Costs and QALYs were both discounted at 3% [14], [28].

Univariate sensitivity analysis was performed by varying single-parameter values according to credible ranges, informed by existing literature, and re-running the model. Parameters selected for further investigation were those most likely to influence differences in cost; probability and cost of job turnover, daily wage and annual salary. Daily wage, weekly wage, and annual salary were replaced with identical values for blue- and white-collar workers to explore whether observed differences between the groups were due to white-collar workers’ higher mean wage. Job turnover cost, represented in both models by a large range of 0.75–1.5 times a worker's annual salary, was widened further to represent the full range of estimates reported in the literature; 0.5–10 times a worker's annual salary. Job turnover probability for workers experiencing depression was increased from 10.5% to 25% and 50%, a realistic probability according to current estimates.

## Results

### Base Case Model

Table 1 presents mean costs and health outcomes for absenteeism and presenteeism over one-year with 95% credible intervals. Outcomes are presented for the base case, and blue- and white-collar models. The 1-year total mean cost of absenteeism per employee with depression was $9626, presenteeism costs were $7864. While total costs and all cost contributors (job turnover costs, lost productive time, antidepressant medication and service use) were higher for absenteeism reporters than presenteeism, the only significant difference was for service use costs (Table 2). One-year health outcomes did not significantly differ for absenteeism reporters relative to presenteeism reporters, based on overlapping 95% credible intervals (Table 1).

**Table 1:** One-year cost and health outcomes of absenteeism and presenteeism.

	Absenteeism		Presenteeism	
	Estimate	95% Credible Interval	Estimate	95% Credible Interval
Blue Collar				
Cost ($ AUD)	6223	4722–5997	5370	5589–6833
QALYs	0.57	0.51–0.60	0.65	0.58–0.66
White Collar				
Cost ($ AUD)	12 938	11 442–14 416	11 178	9662–12 764
QALYs	0.54	0.49–0.56	0.66	0.60–0.71
Base Case				
Cost ($ AUD)	9626	6224–11 384	7864	4452–9565
QALYs	0.60	0.40–0.84	0.68	0.48–0.89

**Table 2:** One year costs of absenteeism and presenteeism.

	Absenteeism		Presenteeism	
	Cost ($ AUD)	95% CIs	Cost ($ AUD)	95% CIs
Blue Collar				
Lost Productive Time	2738	2693–2741	1762	1731–1764
Job Turnover	3456	2899–4055	3586	2964–4173
Service Use	2	1–4	0.16	0.06–0.24
Antidepressants	36	7–46	20	6–45
Total	6223	6326–8048	5370	4522–5952
White Collar				
Lost Productive Time	4070	3995–4075	2198	2158–2201
Job Turnover	8745	7225–10138	8880	7330–10 431
Service Use	16	6–32	4	2–5
Antidepressants	106	104–106	87	85–88
Total	12 938	11 442–14 416	11 178	9662–12764
Base Case				
Lost Productive Time	3095	1945–4457	2032	1308–2680
Job Turnover	6305	3633–7783	5692	2518–7358
Service Use	45	20–62	11	8–14
Antidepressants	179	124–172	128	112–153
Total	9626	6224–11 384	7864	4452–9565

Five-year total simulated costs per worker for absenteeism were $42 573 (95% CI: $19 269–$69 348), presenteeism costs ($37 791, 95% CI $17 475–$66 781) were not significantly different. Further, 5-year health outcomes did not significantly differ for absenteeism (2.70 QALYS; 95% CI 2.16–3.57) compared to presenteeism reporters (3.14 QALYs; 95% CI 2.34–3.75).

### Blue- and White-collar Models

One-year total simulated cost of absenteeism was $6223 per blue-collar worker and $12 938 per white-collar worker (Table 1). Over 1-year, presenteeism cost an estimated $5370 per blue-collar worker and $11 178 per white-collar worker. Job turnover, lost productive time, antidepressant medication and depression-related service use costs were all significantly higher for white-collar workers (Table 2).

While not significantly different, blue-collar workers reporting absenteeism showed a trend towards better 1-year health outcomes (0.57 QALYs; 95% CI 0.51–0.60) compared to white-collar absenteeism reporters (0.54 QALYs; 95% CI 0.49–0.56). Similarly, blue-collar presenteeism reporters had slightly better health outcomes (0.65 QALYs; 95% CI 0.58–0.66) when compared to white-collar presenteeism reporters (0.66 QALYs; 95% CI 0.60–0.71) (Table 1). Within the white collar workforce, presenteeism reporters had significantly better quality of life (0.66 QALYs) compared to white collar workers who reported absenteeism (0.54 QALYs). There were no differences in health outcomes for blue collar workers reporting absenteeism versus presenteeism.

The simulated costs and health outcomes of absenteeism and presenteeism over 5-years differed for blue- versus white-collar workers. Five-year total simulated cost of absenteeism was $26 401 per blue-collar worker and $63 771 per white-collar worker. Presenteeism costs were estimated at $23 711 per blue-collar and $54 709 per white-collar worker. Five-year health outcomes did not significantly differ for blue-collar workers reporting absenteeism (2.74 QALYs) compared to white-collar absenteeism reporters (2.64 QALYs). Nor did they differ for blue-collar presenteeism reporters (3.12 QALYs) when compared to white-collar presenteeism reporters (3.20 QALYs).

### Sensitivity Analysis

Probabilistic sensitivity analysis revealed wide 95% credible intervals around the cost of job turnover for blue-collar and white-collar workers, by absenteeism and presenteeism, for both time frames (Table 2). This highlights the importance of job turnover in terms of its contribution to the overall cost of the models and the need for a more robust estimate.

Base case results revealed 1-year absenteeism costs ranged from $7376–$46 273 per worker (Table 3). In the occupation-specific models 1-year absenteeism costs of ranged from $5075–$22 186 per blue-collar worker, and $9783–$57 740 per white-collar worker (Table 3). Over 1-year, base case presenteeism costs varied from $5500–$46 377. Further, presenteeism costs ranged from $4349–$19 864 per blue-collar worker, and $9783–$55 672 per white-collar worker (Table 3). Using a uniform daily wage and annual salary estimates revealed differences in cost outcomes between blue- and white-collar workers remained i.e. total cost was higher for white-collar workers. Varying cost of job turnover estimates for workers with and without depression symptoms had the most substantial impact on total cost outcomes (Table 4).

**Table 3:** Absenteeism and presenteeism cost outcomes of selected one-way sensitivity analysis over one-year.

		Base Case	
		Presenteeism	Absenteeism
		$/QALY	$/QALY
	Alternative Parameters	7864	9626
Probability of Job Turnover			
Not Depressed	0.025		
	0.05	11 899	13 464
	0.075	12 627	13 530
Depressed	0.105		
	0.25	9886	12 796
	0.50	13 351	18 230
Cost of Job Turnover			
Not Depressed	512	5500	7376
	1154		
	11 615	46 377	46 271
Depressed	2080	7061	8634
	4685		
	47 318	20 997	25 674

**Table 4:** Absenteeism and presenteeism cost outcomes of selected one-way sensitivity analysis over one-year.

		Blue Collar			White Collar	
		Presenteeism	Absenteeism		Presenteeism	Absenteeism
		$/QALY	$/QALY		$/QALY	$/QALY
	Alternative Parameters	5370	6223	Alternative Parameters	11 178	12 938
Probability of Job Turnover						
Not Depressed	0.01			0.025		
	0.02	7 170	8036	0.05	16 533	18 331
	0.05	12 627	13 530	0.06	18 733	20 545
Depressed	0.105			0.105		
	0.25	7823	8474	0.25	15 728	17 235
	0.50	12 019	12 332	0.50	23 527	24 598
Cost of Job Turnover						
Not Depressed	223	4349	5195	717	8044	9783
	502			1614		
	4470	19 864	20 818	14 348	55 672	57 740
Depressed	2303	4395	5075	2912	9373	10 843
	5183			6553		
	46 079	19 167	22 186	38 254	26 889	31 478
Income						
Mean Annual Salary	44 252			55 595		
	72 517	7660	8431	72 517	13 811	15 529
Daily Wage	170			215		
	279	6506	7988	279	11 839	14 162

## Discussion

The purpose of this analysis was to estimate the costs and health outcomes of absenteeism versus presenteeism for employed Australians experiencing major depression, and quantify variations across occupation. Within blue and white-collar groups, absenteeism reporters incurred significantly higher lost productive time, service use, and antidepressant medication costs than presenteeism reporters. Differences in lost productive time costs may be attributable to absenteeism reporters taking time off work due to more severe symptoms, which may also account for their greater antidepressant medication and service use, and the related costs. These findings suggest to employers and health professionals that absenteeism reporters should be the more immediate focus of any health promotion strategies implemented in the workplace.

However, whilst presenteeism costs were often lower than absenteeism costs, they were also substantial, thus indicating the need to better manage this behavior to prevent depression-related productivity loss. Although, not significant, the higher QALYs of employees reporting presenteeism suggests they may be milder depression cases, and their work capacity is reduced but not eliminated. Therefore, employers and health professionals could collaborate to rearrange job tasks to suit employees' abilities [1], and/or provide flexible work attendance arrangements to make the most of employees' work capacity whilst allowing time off when productivity contributions are more severely affected.

Graded sickness absence, which allows employees to work part-time, work full-time hours but perform modified tasks, or perform regular tasks with reduced input whilst receiving a partial sick leave pay and partial salary [29], has been proven effective at keeping people with reduced work ability in working life [30], [31]. Such an approach may have positive effects on health and well-being through the maintenance of their daily routines, and by providing a sense of purpose and opportunities for social support from co-workers. Recognition of reduced capacity may also alleviate stress on the affected worker and improve relationships with co-workers by enabling better planning of how tasks may need to be allocated. To ensure the efficacy of such programs, complementary efforts to reduce stigma associated with mental health issues are required as modifying duties or work-time arrangements may expose employees to the negative effects of stigma and exacerbate their condition [32].

As lost productive time was valued on the basis of mean wage, wage differences between blue- and white- collar workers partly account for the differences by occupational type in overall costs. When a white-collar worker reports depression-related absenteeism or presenteeism the ensuing productivity loss is greater as their time is valued more highly within the labour market. Whilst sensitivity analyses revealed a higher mean wage does not entirely explain the observed differences, it explains the work-related variation, and demonstrates to managers and policy makers the importance of tailoring workplace intervention and promotion strategies to specific occupation types. In particular, employers of white-collar workers, particularly those with paid sick leave entitlements, for whom reducing depression-related absenteeism and presenteeism within this group would have significant cost-saving potential. However, as wages fully explain the aforementioned differences in work-related costs, from a workplace perspective, strategies designed to ameliorate depression-related absenteeism and presenteeism amongst blue-collar workers are equally important.

Higher service use and antidepressant medication costs for white-collar workers may be partly explained by the sex distribution between occupation types in our sample. That is, the combination of women being more likely to disclose depression symptoms and seek treatment [33], and the fact that 85% of females in our sample were white-collar workers, may have increased service-related costs within the white-collar group. This is relevant for managers and employers with a large proportion of female staff, such as those operating in the retail, education, or health sectors. However, these are societal costs and workplace mental health support could have broader benefits beyond specific organisations or work settings. Therefore, investment in the mental health and wellbeing of the workforce should be seen as priority for society in general as well employers.

Disparate health outcomes between occupation groups, suggest depression and related work attendance decisions affect blue and white-collar workers differently. White-collar absenteeism reporters experienced poorer QALYs than their blue-collar counterparts and depression-related absenteeism *and* presenteeism costs were higher for white collar workers. This may help to identify areas of priority in regards to mental health promotion and prevention. In particular, the costs associated with absenteeism for white-collar workers, borne by employers via lost productive time and by employees via service use and antidepressant medication costs, suggest they are an important focus of future workplace health promotion strategies with the potential to deliver individual and societal benefits.

### Limitations

Lower QALYs amongst absenteeism compared to presenteeism reporters may be due to absenteeism reporters experiencing more severe symptoms which restrict their work ability and impact quality-of-life. However, whether presenteeism reporters have higher QALYs due to benefits of continued work attendance, or whether continuing to work is due to higher quality-of-life, remains unclear. Analysis of absenteeism versus presenteeism costs and health outcomes stratified by severity of depression may allow recommendations as to whether presenteeism is advisable, and whether absenteeism reporters should be encouraged to return to work promptly. The inability to source each model input stratified by depression severity status precluded such an analysis being conducted and was one of this study's major limitations.

Further, job turnover was the largest contributor to overall cost, but sensitivity analysis revealed the probability and cost estimates for job turnover used had substantial uncertainty around them; 95% credible intervals were wide. Additionally, potentially relevant costs, including those attributable to depression-related workplace accidents, were excluded due to inability to find a reliable estimate which met established quality of evidence criteria [22]. Exploratory sensitivity analysis revealed workplace accidents costs contributed substantially to total cost and future effort should be directed at understanding the magnitude of this problem.

Presenteeism behaviour was defined in the initial scenarios as the absence of depression-related absenteeism in the 12-months prior to the NSMHWB interview. This initial classification assumes that the categories of 12-month absenteeism and presenteeism were mutually exclusive, which is not always correct. Recent research has identified that episodes of absenteeism are often preceded and followed by episodes of presenteeism [38]. This highlights that employed individuals reporting depression can experience both absenteeism and presenteeism within a given period of time. Therefore there may be some error in classification at baseline based on this definition. However, while there was a NSMHWB item which asked respondents to report the number disability, or presenteeism, days they experienced in the past year, separate to an item which asked individuals to report their number of absence days, it was not depression specific. Therefore, although the method we employed to define presenteeism may be considered a limitation, it was depression-specific and removed the possible influence of co-morbid disorders on work attendance decisions. Further, as we modelled what happened to our hypothetical cohort in 3-month cycles, individuals were assigned probabilities of lost productive time based on both absenteeism and presenteeism as we know they move in and out of these states over time.

Absenteeism reporters had lower QALYs, albeit not significantly, compared to presenteeism reporters. This may be due to individuals reporting absenteeism experiencing more severe symptoms which restrict their work ability and by extension their quality-of-life. However, what remains unclear is whether individuals reporting presenteeism have higher quality-adjusted life years due to benefits of continued work attendance such as social support, structured routine and income or whether continuing to work is due to higher quality-of-life. This highlights the need for longitudinal data examining the impact of continued work attendance not only on Quality of Life amongst employed individuals reporting depression, but also whether any observed changes are as a result of their changes in the severity of their depression. Such data would enable further exploration s of absenteeism versus presenteeism costs and health outcomes stratified by severity of depression, and may allow recommendations as to whether continuing to work is advisable and whether absenteeism reporters should be encouraged to return to work as soon as possible. The inability to source individual model inputs stratified by depression severity status precluded such an analysis being conducted in the present study.

### Strengths

This study's most notable strength was the use of a quality epidemiological data source providing representative estimates of the Australian working population [15]. This allows generalizability of our findings and facilitates their translation to all employed Australians. Another strong point is the major depression diagnoses provided by the NSMHWB, determined using the modified version of the World Mental Health Survey Initiative version of the Composite International Diagnostic Interview (WMH-CIDI). This instrument has undergone extensive methodological testing and development which ensures the international comparability of our results. Additionally, occupation type is an objectively measured variable which eliminates the potential for answers regarding working characteristics to be influenced by response style (acquiescence, social desirability), personality characteristics and negative affect [34]; an important consideration within a sample of individuals experiencing depression.

### Conclusion

These findings could inform workplace health promotion strategies aimed at improving the management of depression and related work attendance behaviour, and benefit employees, employers and broader society via investment in a healthy and productive workforce. Informing employers and health care professionals of the health *and* economic benefits of presenteeism for employees experiencing depression could encourage them to adapt work environments, allow employees to perform modified tasks, and offer flexible work time arrangements to promote continued work attendance [29]. Such action may decrease productivity loss, as employers use their employees' remaining work ability more effectively, and reduce turnover and employee replacement costs as employees with depression continue to be productive members of the workforce [30]. Secondly, and of interest to health professionals, such workplace modifications may have positive, long-term effects on health and well-being via maintained daily routine and co-worker support. Finally, the exploration of these outcomes by occupation type allows work attendance recommendations to be tailored to specific occupation types. Such information may be of particular importance for specific occupations or sectors with strong attendance demands such as small businesses, who lack the human capital to compensate for the lost productivity associated with absenteeism, or health care professionals with difficult to substitute skills.

## Supporting Information

Table S1 **Data inputs and assumptions in base case model.**
(DOCX)

Table S2 **Data inputs and assumptions in absenteeism model.**
(DOCX)

Table S3 **Data inputs and assumptions in presenteeism model, where estimates differ from absenteeism model.**
(DOCX)

Table S4 **Absenteeism and presenteeism days and associated lost productive time by occupation type.**
(DOCX)

## References

[1] Sanderson K, Tilse E, Nicholson J, Oldenburg B, Graves N (2007) Which presenteeism measures are more sensitive to depression and anxiety? Journal of Affective Disorders 101: 65–74. 17156851 10.1016/j.jad.2006.10.024

[2] Stewart WF, Ricci JA, Chee E, Hahn SH, Morganstein D (2003) Cost of lost productive work time among US workers with depression. Journal of the American Medical Association 289: 3135–3144. 12813119 10.1001/jama.289.23.3135

[3] Sanderson K, Andrews G (2006) Common mental disorders in the workforce: recent findings from descriptive and social epidemiology. Canadian Journal of Psychiatry 51: 63–75. 16989105 10.1177/070674370605100202

[4] Stewart WF, Ricci JA, Chee E, Hahn SR, Morganstein D (2003) Cost of lost productive work time among US workers with Depression. JAMA 289: 3135–3144. 12813119 10.1001/jama.289.23.3135

[5] Cox A, Ness K, Carlson R (2010) International perspectives on depression in the workplace.

[6] LaMontagne A, Sanderson K, Cocker F (2010) Estimating the economic benefits of eliminating job strain as a risk factor for depression. Melbourne, Australia: Victorian Health Promotion Foundation (VicHealth). 10.1097/JOM.0000000000000908 28045792

[7] Aronsson G, Gustafsson K (2005) Sickness presenteeism: Prevalence, attendance-pressure factors, and an outline of a model for research. JOEM 47: 958–966. 16155481 10.1097/01.jom.0000177219.75677.17

[8] Kivimaki M, Head J, Ferrie JE, Hemingway H, Shipley MJ, et al (2005) Working while ill as a risk factor for serious coronary events: The Whitehall II Study. American Journal of Public Health 95: 98–100. 15623867 10.2105/AJPH.2003.035873 PMC1449859

[9] Bergstrom G, Bodin L, Hagberg J, Lindh T, Aronsson G, et al (2009) Does sickness presenteeism have an impact of future general health? Int Arch Occup Environ Health 82: 1179–1190. 19504117 10.1007/s00420-009-0433-6

[10] Bergstrom G, Bodin L, Hagberg J, Aronsson G, Josephson M (2009) Sickness presenteeism today, sickness absenteeism tomorrow? A prospective study in sickness presenteeism and future sickness absenteeism. JOEM 51: 629–638. 19448572 10.1097/JOM.0b013e3181a8281b

[11] Väänänen A, Toppinen-Tannera S, Kalimoa R, Mutanenc P, Vahterad J, et al (2003) Job characteristics, physical and psychological symptoms, and social support as antecedents of sickness absence among men and women in the private industrial sector. Social Science & Medicine 57: 807–824. 12850108 10.1016/s0277-9536(02)00450-1

[12] Aronsson G, Gustafsson K, Dallner M (2000) Sick but yet at work. An empirical study of sickness presenteeism. J Epidemiol Community Health 54: 502–509. 10846192 10.1136/jech.54.7.502 PMC1731716

[13] Hilton MF, Scuffham P, Sheridan JS, Cleary CM, Whiteford HA (2008) Mental ill-health and the differential effect of employee type on absenteeism and presenteeism. Journal of Occupational and Environmental Medicine 50: 1228–1243. 19001949 10.1097/JOM.0b013e31818c30a8

[14] Wang PS, Patrick A, Avorn J, Azocar F, Ludman E, et al (2006) The costs and benefits of enhanced depression care to employers. Archives of General Psychiatry 63: 1345–1353. 17146009 10.1001/archpsyc.63.12.1345

[15] Australian Bureau of Statistics (2007) National Survey of Mental Health and Wellbeing (Cat. No. 4326.0), Basic CURF. Canberra, ACT: Australian Bureau of Statistics.

[16] Australian Bureau of Statistics (2006) The Australian and New Zealand Standard Classification of Occupations (ANZSCO) First Edition (cat. no. 1220.0). Canberra, ACT: ABS.

[17] Lerner D, Adler DA, Chang H, Lapitsky L, Hood MY, et al (2004) Unemployment, job retention, and productivity loss among employees with depression. Psychiatric Services 55: 1371–1378. 15572564 10.1176/appi.ps.55.12.1371 PMC4283817

[18] Australian Federal Department of Education Employment and Workplace Relations (2008) Evaluating work and family strategies in your workplace. In: Relations AFDoEEaW, editor. Canberra:ACT: Commonwealth of Australia.

[19] Simon GE, Revicki D, Heiligenstein J, Grothaus L, VanKorff M, et al (2000) Recovery from depression, work productivity, and health care costs among primary care patients. General Hospital Psychiatry 22: 153–162. 10880708 10.1016/s0163-8343(00)00072-4

[20] Rost K, Smith JL, Dickinson M (2004) The effect of improving primary care depression management on employee absenteeism and productivity: A randomised trial. Medical Care 42: 1202–1210. 15550800 10.1097/00005650-200412000-00007 PMC1350979

[21] Teesson M, Slade T, Mills K (2009) Comorbidity in Australia: findings of the 2007 National Survey of Mental Health and Wellbeing. Australian and New Zealand Journal of Psychiatry 43: 606–614. 19530017 10.1080/00048670902970908

[22] Braithwaite S, Roberts MS, Justice AC (2007) Incorporating quality of evidence into decision analytic modelling. Ann Int Med 146: 133–141. 17227937 10.7326/0003-4819-146-2-200701160-00008 PMC3460380

[23] Therapeutic Guidelines Limited. Psychotropic Writing Group (2008) Therapeutic Guidelines: psychotropic; Dowden J, editor. Melbourne: Therapeutic Guidelines Limited.

[24] Sanderson K, Andrews G, Corry J, Lapsley H (2004) Modelling change in preference values from descriptive health status using the effect size. Quality of Life Research 13: 1255–1264. 15473504 10.1023/B:QURE.0000037482.92757.82

[25] Hawthorne G, Richardson J, Osborne R (1999) The Assessment of Quality of Life (AQoL) instrument: a psychometric measure of health-related quality of life. Quality of Life Research 8: 209–224. 10472152 10.1023/a:1008815005736

[26] Hawthorne G, Osborne R (2005) Population norms and meaningful differences for the Assessment of Quality of Life (AQoL) measure. Blackwell Publishing Ltd. pp. 136–142. 10.1111/j.1467-842x.2005.tb00063.x 15915617

[27] Briggs AH (2000) Handling uncertainty in cost-effectiveness models. Pharmacoecon 17: 479–500. 10.2165/00019053-200017050-00006 10977389

[28] Gold M, Siegel J, Russell L, Weinstein MC, editors (1996) Cost effectiveness in Health and Medicine. New York, NY: Oxford University Press.

[29] Kausto J, Miranda H, Martimo KP, Viikari-Juntura E (2008) Partial sick leave - review of its use, effects and feasibility in the Nordic countries. Scand J Work Environ Health 34: 239–249. 18815712 10.5271/sjweh.1266

[30] Kausto J, Virta L, Luukkonen R, Viikari-Juntura E (2010) Associations between partial sickness benefit and disability pensions: initial findings of a Finnish nationwide register study. BMC Public Health 10: 1–11. 20573207 10.1186/1471-2458-10-361 PMC2912806

[31] Markussen S, Mykletun A, Røed K (2010) The Case for Presenteeism. Oslo, Norway: Ragnar Frisch Centre for Economic Research and IZA Norwegian Institute of Public Health.

[32] Martin A (2010) Individual and contextual correlates of managers' attitudes toward depressed employees. Human Resource Management 49: 647–668.

[33] Burgess PM, Pirkis JE, Slade TN, Johnston AK, Meadows GN, et al (2009) Service use for mental health problems: findings from the 2007 National Survey of Mental Health and Wellbeing. Aust NZ J of Psychiatry 43: 615–623. 10.1080/00048670902970858 19530018

[34] Birnbaum HG, Leong SA, Greenberg PE (2003) The economics of women and depression: an employer's perspective. J Affect Disorders 74: 15–22. 12646295 10.1016/s0165-0327(02)00427-5

[35] Spitzer RL, Williams JW, Kroenke K (1994) Utility of a new procedure for diagnosing mental disorders in primary care: The prime-md 1000 study. JAMA 272: 1749–56. 7966923

[36] Loerch B, Szegedi A, Kohnen R, Benkert O (2000) The primary care evaluation of mental disorders (PRIME-MD), German version: a comparison with the CIDI. J Psych Res 34: 211–20. 10.1016/s0022-3956(00)00005-4 10867116

[37] Kruijshaar ME, Barendregt J, Vos T, de Graaf R, Spijker J (2005) Lifetime prevalence estimates of major depression: An indirect estimation method and a quantification of recall bias. Euro J Epi 20: 103–11. 10.1007/s10654-004-1009-0 15756910

[38] Leineweber C, Westerlund H, Hagberg J, Svedberg P, Alexanderson K (2012) Sickness presenteeism is more than an alternative to sickness absence: results from the population-based SLOSH study. Int Arch Occup Env Health 85: 905–14. 22270388 10.1007/s00420-012-0735-y

