# Supplementary material for: Depression in Working Adults: Comparing the Costs and Health Outcomes of Working When Ill
Source: PLoS One. 2014 Sep 2;9(9):e105430. doi: 10.1371/journal.pone.0105430 (PMC4152191; doi:10.1371/journal.pone.0105430)
Supplement: Table S1 — Data inputs and assumptions in base case model. (DOCX) [file pone.0105430.s001.docx]

|  | Absenteeism |  | Presenteeism |  |  |
| --- | --- | --- | --- | --- | --- |
| Variable/Parameter | Mean | Distribution/Range | Mean | Distribution/Range | Source |
| Initial Probabilities - Health States |  |  |  |  |  |
| Depressed, treatment | 0.208 | 0.202 - 0.214 | † | † | NSMHWB2^Ω^ |
| Depressed, no treatment | 0.167 | 0.162 - 0.172 | † | † | NSMHWB2 |
| Recovered, treatment | 0.107 | 0.104 - 0.110 | † | † | NSMHWB2 |
| Recovered, no treatment | 0.517 | 0.502 - 0.533 | † | † | NSMHWB2 |
|  |  |  |  |  |  |
| Transition Probabilities |  |  |  |  |  |
| Age | 40.70 | Normal (α 40.7, σ 12.76) | † | † | NSMHWB2 |
| Mortality | 0.011 | 0.0003-0.078 | † | † | (1) |
| Suicide, Depressed | 0.0002 | 0.000202-0.000247 | † | † | [(2)](#_ENREF_17) |
| Treatment initiation – Depressed | 0.19 | Beta (α 405.93, β 1730.57) | 0.0117 | Beta (α 9.86, β 852.2) | [(2)](#_ENREF_17) |
| Treatment drop out – Depressed | 0.12 | Beta (α 17.81, β131.17) | † | † | [(2)](#_ENREF_17) |
| Treatment drop out – Recovered | 0.48 | Beta (α 70.95, β 83.29) | † | † | [(2)](#_ENREF_17) |
| Relapse – Recovered, treatment | 0.12 | Beta (α 10.23, β 73.95) | † | † | [(2)](#_ENREF_17), [(3)](#_ENREF_64) |
| Relapse – Recovered, no treatment | 0.25 | Beta (α 0.38, β 1.09) | † | † | [(2)](#_ENREF_17), [(4)](#_ENREF_65) |
| Remission – Treatment | 0.55 | Beta (α 39.1, β 31.83) | † | † | [(2)](#_ENREF_17) |
| Remission – No treatment | 0.13 | Beta (α 24.28, β 154.76) | † | † | [(2)](#_ENREF_17) |
|  |  |  |  |  |  |
| Miscellaneous Probabilities |  |  |  |  |  |
|  |  |  |  |  |  |
| 3-mo GP visits | 0.085 | Beta (α 481.51, β 5161.47) | 0.047 | Beta (α 78.78, β 1599.7) | NSMHWB2 |
| Depressed, treatment | 0.067 | Beta (α 157.08, β 2171.75) | 0.037 | Beta (α 48.72, β 1273.7) | NSMHWB2 |
| Depressed, no treatment | 0.013 | Beta (α 119.12, β 8602.1) | 0.008 | Beta (α 96.9, β 11919.9) | NSMHWB2 |
| Recovered, treatment | § | § | § | § |  |
| Recovered, no treatment | § | § | § | § |  |
| Early Retired | 0.285 | Beta (α 265.2, β 737.2) | 0.118 | Beta (α 58.4, β 437.7) | NSMHWB2 |
| Retired | 0.356 | Beta (α 451.3, β 827.9) | 0.086 | Beta (α 481.51, β 4761.47) | NSMHWB2 |
|  |  |  |  |  |  |
| 3-mo psychiatrist visits | 0.015 | Beta (α 359.57, β 26274.93) | 0.007 | Beta (α 114.39, β 16164.11) | NSMHWB2 |
| Depressed, treatment | 0.012 | Beta (α 505.65, β 39492.85) | 0.007 | Beta (α 114.39, β 16164.11) | NSMHWB2 |
| Depressed, no treatment | § | § | § | § | NSMHWB2 |
| Recovered, treatment | § | § | § | § |  |
| Recovered, no treatment | § | § | § | § |  |
| Early Retired | 0.062 | Beta (α 259.62, β 3871.32) | 0.026 | Beta (α 31.51, β 1278.9) | NSMHWB2 |
| Retired | 0.063 | Beta (α 263.72, β 3869.24) | § | § | NSMHWB2 |
|  |  |  |  |  |  |
| 3-mo psychologist visits | 0.029 | Beta (α 1389.8, β 44996.8) | 0.0205 | Beta (α 998.7, β 48898.4) | NSMHWB2 |
| Depressed, treatment | 0.033 | Beta (α 1316.4, β 38527.1) | 0.0205 | Beta (α 998.7, β 48898.4) | NSMHWB2 |
| Depressed, no treatment | § | § | § | § | NSMHWB2 |
| Recovered, treatment | § | § | § | § |  |
| Recovered, no treatment | § | § | § | § |  |
| Early Retired | 0.062 | Beta (α 259.62, β 3871.32) | 0.071 | Beta (α 372.42, β 4765.12) | NSMHWB2 |
| Retired | 0.034 | Beta (α 1589.7, β 43982.4) | § | § | NSMHWB2 |
|  |  |  |  |  |  |
| Number of consults |  |  |  |  |  |
| General practitioner (GP) | 2.5 | Uniform (Low=1, High=4) | † | † | NSMHWB2 |
| Psychiatrist | 2.5 | Uniform (Low=1, High=4) | † | † | NSMHWB2 |
| Psychologist | 9 | Uniform (Low=6, High=12) | † | † | NSMHWB2 |
|  |  |  |  |  |  |
| 3-mo antidepressant use |  |  |  |  |  |
| Total | 0.690 | Beta (α 166.2, β 72.25) | 0.325 | Beta (α 8.58, β 17.93) | NSMHWB2 |
| Depressed, treatment | 1.055 | Normal (σ 0.072) | 0.852 | Beta (α 78.26, β 13.499) | NSMHWB2 |
| Depressed, no treatment | 0.160 | Beta (α 444.6, β 2283.0) | 0.052 | Beta (α 320.37, β 5840.63) | NSMHWB2 |
| Recovered, treatment | 2.241 | Normal (σ 0.094) | 1.692 | Normal (σ 0.084) | NSMHWB2 |
| Recovered, no treatment | 0.348 | Beta (α 394.45, β 739.03) | 0.106 | Beta (α 567.89, β 4739.5) | NSMHWB2 |
| Early Retired | 1.23 | Normal (σ 0.081) | § | § | NSMHWB2 |
| Retired | 0.80 | Beta (α 216.81, β 52.43) | 1.41 | Normal (σ 0.079) | NSMHWB2 |
|  |  |  |  |  |  |
| Job Turnover |  |  |  |  |  |
| Depressed | 0.104 | Beta (α 538.29, β 4637.60) | † | † | [(5)](#_ENREF_19) |
| Recovered | 0.025 | Beta (α 1267.31, β 48431.19) | † | † | [(5)](#_ENREF_19) |
|  |  |  |  |  |  |
| Absenteeism Days |  |  |  |  |  |
| Depressed, treatment | 14.02 | 95% CI: 10.1 - 17.7 | **§** | **§** | NSMHWB2 |
| Depressed, no treatment | 5.5 | 95% CI: 3.0 - 8.3 | **§** | **§** | NSMHWB2 |
| Recovered, treatment | 1.6 | 95% CI: 0.1 - 2.7 | **§** | **§** | NSMHWB2 |
| Recovered, no treatment | 1.9 | 95% CI: 0.2 - 3.5 | **§** | **§** | NSMHWB2 |
|  |  |  |  |  |  |
| Presenteeism Days |  |  |  |  |  |
| Depressed, treatment | **§** | **§** | 3.3 | 95% CI: 0.9 - 5.6 | NSMHWB2 |
| Depressed, no treatment | **§** | **§** | 4.2 | 95% CI: 1.1 - 7.7 | NSMHWB2 |
| Recovered, treatment | **§** | **§** | 1.7 | 95% CI: 0.6 - 2.8 | NSMHWB2 |
| Recovered, no treatment | **§** | **§** | 2.2 | 95% CI: 0.1 - 3.9 | NSMHWB2 |
|  |  |  |  |  |  |
| Costs |  |  |  |  |  |
| Daily Wage | 176 | 149-202 | † | † | [(6)](#_ENREF_66) |
| Weekly Wage | 880 | Gamma (α 8586.46, β 9.75) | † | † | [(6)](#_ENREF_66) |
| Annual Salary | 39999.00 | Gamma (α 172.98, β 0.0044) | † | † | [(6)](#_ENREF_66) |
| Daily Hours | 6.9 | 95% CI: 5.2 - 8.7 | † | † | [(6)](#_ENREF_66) |
| Weekly Hours | 34.4 | 95% CI: 32.4 - 36.5 | † | † | [(6)](#_ENREF_66) |
|  |  |  |  |  |  |
| Lost Productive Time |  | 25% Range |  | 25% Range |  |
| Depressed, treatment | 2469 | 1852 - 3087 | 573 | 430 - 717 | [(7)](#_ENREF_2), [(8)](#_ENREF_22), [(9)](#_ENREF_21) |
| Depressed, no treatment | 963 | 723 - 1205 | 736 | 553 - 921 | [(7)](#_ENREF_2), [(8)](#_ENREF_22), [(9)](#_ENREF_21) |
| Recovered, treatment | 277 | 208 - 347 | 300 | 225 - 375 | [(7)](#_ENREF_2), [(8)](#_ENREF_22), [(9)](#_ENREF_21) |
| Recovered, no treatment | 340 | 255 - 426 | 388 | 292 - 486 | [(7)](#_ENREF_2), [(8)](#_ENREF_22), [(9)](#_ENREF_21) |
|  |  |  |  |  |  |
| Job Turnover |  | 25% Range |  | 25% Range |  |
| Depressed, treatment | 4685 | 3514 - 5857 | † | † | [(5)](#_ENREF_19), [(6)](#_ENREF_66) |
| Depressed, no treatment | 1154 | 866 - 1443 | † | † | [(5)](#_ENREF_19), [(6)](#_ENREF_66) |
| Recovered, treatment | 1154 | 866 - 1443 | † | † | [(5)](#_ENREF_19), [(6)](#_ENREF_66) |
| Recovered, no treatment | 1154 | 866 - 1443 | † | † | [(5)](#_ENREF_19), [(6)](#_ENREF_66) |
|  |  |  |  |  |  |
| Service Use |  |  |  |  |  |
| 3-mo Antidepressant Use | 52.33 | 44.5 - 60.2 | 24.63 | 20.9 - 28.3 | NSMHWB2, [(10)](#_ENREF_37), [(11)](#_ENREF_67) |
| Depressed, treatment | 79.96 | 68.0 - 92.0 | 64.59 | 54.9 - 74.3 |  |
| Depressed, no treatment | 12.14 | 10.3 - 14.0 | 3.99 | 3.4 - 4.6 |  |
| Recovered, treatment | 169.87 | 144.4 - 195.4 | 128.26 | 109.0 - 147.5 |  |
| Recovered, no treatment | 26.42 | 22.5 - 30.4 | 8.09 | 6.9 - 9.3 |  |
| Early Retired | 93.24 | 79.3 - 107.2 | § | § |  |
| Retired | 60.8 | 51.7 - 69.9 | 107.43 | 91.3 - 123.5 |  |
|  |  |  |  |  |  |
| GP visit (>5 < 25 mins) | 22.22 | 20.0 - 24.20 | † | † | NSMHWB2, [(10)](#_ENREF_37), [(11)](#_ENREF_67), (12, 13) |
| Depressed, in treatment | 4.52 | 3.8 - 5.2 | 1.66 | 1.4 - 1.9 |  |
| Depressed, no treatment | 0.61 | 0.5 - 0.7 | 0.18 | 0.15 - 0.21 |  |
| Recovered, treatment | § | § | § | § |  |
| Recovered, no treatment | § | § | § | § |  |
| Early Retired | 6.33 | 5.4 - 7.3 | 2.63 | 2.2 - 3.0 |  |
| Retired | 7.92 | 6.7 - 9.1 | 1.92 | 1.6 - 2.2 |  |
|  |  |  |  |  |  |
| Psychiatrist visit (>30 <45 mins) | 108.12 | 97.3 - 118.93 | † | † | NSMHWB2, [(10)](#_ENREF_37), [(11)](#_ENREF_67), (12, 13) |
| Depressed, in treatment | 3.46 | 2.94 - 3.98 | 2.05 | 1.74 - 2.36 |  |
| Depressed, no treatment | 0.68 | 0.58 - 0.78 | § | § |  |
| Recovered, treatment | § | § | § | § |  |
| Recovered, no treatment | § | § | § | § |  |
| Early Retired | 16.8 | 14.3 - 19.3 | 7.11 | 6.0 - 8.2 |  |
| Retired | 16.9 | 14.4 - 19.5 | § | § |  |
|  |  |  |  |  |  |
| Psychologist visit (>60 mins) | 181.54 | 163.38 - 199.69 | † | † | NSMHWB2, [(10)](#_ENREF_37), [(11)](#_ENREF_67), (12, 13) |
| Depressed, in treatment | 55.00 | 46.7 - 63.2 | 33.61 | 28.5 - 38.6 |  |
| Depressed, no treatment | § | § | § | § |  |
| Recovered, treatment | § | § | § | § |  |
| Recovered, no treatment | § | § | § | § |  |
| Early Retired | 101.95 | 86.6 - 117.2 | 116.36 | 98.9 - 133.8 |  |
| Retired | 55.43 | 46.9 - 63.5 | § | § |  |
|  |  |  |  |  |  |
| Utilities – AQoL^α^ Values |  |  |  |  |  |
| Depressed, treatment | 0.116 | Beta (α 118.8, β 905.6) | 0.148 | Beta (α 110.3, β 634.8) | NSMHWB2 |
| Depressed, no treatment | 0.123 | Beta (α 163.6, β 1167.0) | 0.146 | Beta (α 107.5, β 629.2) | NSMHWB2 |
| Recovered, treatment | 0.133 | Beta (α 425.87, β 2776.2) | 0.170 | Beta (α 374.6, β 1829.0) | NSMHWB2 |
| Recovered, no treatment | 0.156 | Beta (α 2282.0, β 12346.3) | 0.186 | Beta (α 574.5, β 2514.3) | NSMHWB2 |
| Early Retired | 0.093 | Beta (α 68.7, β 685.2) | 0.114 | Beta (α 89.7, β 705.3) | NSMHWB2 |
| Retired | 0.224 | Beta (α 634.1, β 2219.8) | 0.214 | Beta (α 609.2, β 2199.3) | NSMHWB2 |
|  |  |  |  |  |  |
| Discount rate | 3% | 0-5 | † | † | [(2)](#_ENREF_17) |

* National Survey of National Survey of Wellbeing (2007)

†Denotes the same value for each decision option.

‡ Assessment of Quality of Life-4D

§ No data available for this parameter from the 2007 NSMHWB e.g.no service or antidepressant use reported.
