# Supplementary material for: Depression in Working Adults: Comparing the Costs and Health Outcomes of Working When Ill
Source: PLoS One. 2014 Sep 2;9(9):e105430. doi: 10.1371/journal.pone.0105430 (PMC4152191; doi:10.1371/journal.pone.0105430)
Supplement: Table S2 — Data inputs and assumptions in absenteeism model. (DOCX) [file pone.0105430.s002.docx]

| Variable/Parameter | White Collar |  | Blue Collar |  |  |
| --- | --- | --- | --- | --- | --- |
| Initial Probabilities - Health States | Mean | Distribution OR Range | Mean | Distribution OR Range | Source |
| Depressed, treatment | 0.195 | 0.176-0.215 | 0.242 | 0.219-0.267 | 2007 NSMHWB^*^ |
| Depressed, no treatment | 0.159 | 0.143-0.175 | 0.2 | 0.180-0.220 | 2007 NSMHWB |
| Recovered, treatment | 0.116 | 0.105-0.128 | 0.076 | 0.069-0.084 | 2007 NSMHWB |
| Recovered, no treatment | 0.528 | 0.476-0.581 | 0.480 | 0.433-0.529 | 2007 NSMHWB |
|  |  |  |  |  |  |
| Transition Probabilities |  |  |  |  |  |
| Age | 40.61 | Normal (α 40.61, σ 12.70) | 38.4 | Normal (α 38.4, σ 12.95) | 2007 NSMHWB |
| Mortality | 0.011 | 0.0003-0.078 | † | † | [(1)](#_ENREF_63) |
| Suicide, Depressed | 0.0002 | 0.000202-0.000247 | † | † | [(2)](#_ENREF_17) |
| Treatment initiation - Depressed | 0.19 | Beta (α 21.89, β 92.31) | 0.012 | Beta (α 0.6, β 50.79) | [(2)](#_ENREF_17) |
| Treatment drop out – Depressed | 0.12 | Beta (α 17.81, β 131.17) | † | † | [(2)](#_ENREF_17) |
| Treatment drop out – Recovered | 0.48 | Beta (α 13.75, β 14.47) | † | † | [(2)](#_ENREF_17) |
| Relapse – Recovered, treatment | 0.12 | 0.015-0.085 | † | † | [(2)](#_ENREF_17), [(3)](#_ENREF_64) |
| Relapse – Recovered, no treatment | 0.25 | 0.044-0.43 | † | † | [(2)](#_ENREF_17), [(4)](#_ENREF_65) |
| Remission – Treatment | 0.55 | 0.47-0.62 | † | † | [(2)](#_ENREF_17) |
| Remission – No treatment | 0.13 | 0.09-0.15 | † | † | [(2)](#_ENREF_17) |
|  |  |  |  |  |  |
| Miscellaneous Probabilities |  |  |  |  |  |
| 3-mo primary care physician visits | 0.013 | Beta (α 0.64, β 48.48) | 0.015 | Beta (α 1.04, β 68.23) | 2007 NSMHWB |
| 3-mo psychiatrist visits | 0.002 | Beta (α 0.04, β 20.54) | 0.002 | Beta (α 0.05, β 18.28) | 2007 NSMHWB |
| 3-mo psychologist visits | 0.005 | Beta (α 0.13, β 22.90) | 0.007 | Beta (α 0.42, β 57.10) | 2007 NSMHWB |
| Number of GP consults | 2.5 | Uniform (Low=1, High=4) | † | † | 2007 NSMHWB |
| Number of Psychiatrist consults | 2.5 | Uniform (Low=1, High=4) | † | † | 2007 NSMHWB |
| Number of Psychologist consults | 9 | Uniform (Low=6, High=12) | † | † | 2007 NSMHWB |
|  |  |  |  |  |  |
| 3-mo antidepressant use |  |  |  |  |  |
| Total | 0.158 | Beta (α 7.53, β 40.11) | 0.147 | Beta (α 6.93, β 40.11) | 2007 NSMHWB |
| Depressed, treatment | 0.699 | Beta (α 11.46, β 4.90) | 0.221 | Beta (α 8.83, β 31.24) | 2007 NSMHWB |
| Depressed, no treatment | 0.128 | Beta (α 6.57, β 44.79) | 0.03 | Beta (α 1.01, β 31.59) | 2007 NSMHWB |
| Recovered, treatment | 4.605 | Beta (α -942.44, β 737.56) | 0.416 | Beta (α 4.39, β 6.19) | 2007 NSMHWB |
| Recovered, no treatment | 0.251 | Beta (α 21.89, β 65.31) | 0.04 | Beta (α 1.29, β 30.54) | 2007 NSMHWB |
|  |  |  |  |  |  |
| Costs |  |  |  |  |  |
| Daily Wage | 215.18 | Gamma (α 65.8, β 0.31) | 170.20 | Gamma (α 39.6, β 0.23) | [(6)](#_ENREF_66) |
| Weekly Wage | 1075.88 | Gamma (α 8886.46, β 8.25) | 851.00 | Gamma (α 8586.46, β 10.05) | [(6)](#_ENREF_66) |
| Annual Salary | 55945.50 | Gamma (α 218.48, β 0.0039) | 44252.50 | Gamma (α 187.08, β 0.0042) | [(6)](#_ENREF_66) |
| Daily Hours | 7.7 | 95% CI: 7.57-14.9 | 7.8 | 95% CI: 7.56-8.03 | [(6)](#_ENREF_66) |
| Weekly Hours | 38.4 | 95% CI: 37.2-39.6 | 38.9 | 95% CI: 37.8-40.1 | [(6)](#_ENREF_66) |
|  |  |  |  |  |  |
| Lost Productive Time |  |  |  |  |  |
| Depressed, treatment | 3066.32 | 2759.70-3373.0 | 2426.20 | 2183.58-2668.82 | [(7)](#_ENREF_2), [(8)](#_ENREF_22), [(9)](#_ENREF_21) |
| Depressed, no treatment | 1441.70 | 1297.5-1585.9 | 881.21 | 793.08-969.33 | [(7)](#_ENREF_2), [(8)](#_ENREF_22), [(9)](#_ENREF_21) |
| Recovered, treatment | 338.90 | 305.0-375.8 | 251.05 | 225.94-276.15 | [(7)](#_ENREF_2), [(8)](#_ENREF_22), [(9)](#_ENREF_21) |
| Recovered, no treatment | 416.18 | 374.6-457.8 | 312.16 | 280.94-343.37 | [(7)](#_ENREF_2), [(8)](#_ENREF_22), [(9)](#_ENREF_21) |
|  |  |  |  |  |  |
| Job Turnover |  |  |  |  |  |
| Depressed, treatment | 6553.64 | 5898.28-7209.00 | 5183.88 | 4665.49-5702.27 | [(5)](#_ENREF_19), [(6)](#_ENREF_66) |
| Depressed, no treatment | 1614.16 | 1452.74-1775.58 | 502.88 | 419.45-512.67 | [(5)](#_ENREF_19), [(6)](#_ENREF_66) |
| Recovered, treatment | 1614.16 | 1452.74-1775.58 | 502.88 | 419.45-512.67 | [(5)](#_ENREF_19), [(6)](#_ENREF_66) |
| Recovered, no treatment | 1614.16 | 1452.74-1775.58 | 502.88 | 419.45-512.67 | [(5)](#_ENREF_19), [(6)](#_ENREF_66) |
|  |  |  |  |  |  |
| Service Use |  |  |  |  |  |
| 3-mo Antidepressant Use | 25.26 | 22.70-27.80 |  |  | 2007 NSMHWB, [(10)](#_ENREF_37), [(11)](#_ENREF_67) |
| Depressed, treatment | 52.97 | 47.7-58.63 | 16.81 | 15.13-18.49 |  |
| Depressed, no treatment | 9.70 | 8.7-10.7 | 2.29 | 2.06-2.52 |  |
| Recovered, treatment | 75.78 | 68.2-83.36 | 31.56 | 28.40-34.72 |  |
| Recovered, no treatment | 19.03 | 17.1-20.9 | 3.06 | 2.75-3.37 |  |
|  |  |  |  |  |  |
| Primary care physician visit (>5 < 25 mins) | 22.22 | 20.0-24.20 | † | † | 2007 NSMHWB, [(10)](#_ENREF_37), [(11)](#_ENREF_67), (12, 13) |
| Depressed, in treatment | 0.697 | 0.627-0.767 | 0.68 | 0.61-75 |  |
| Depressed, no treatment | 0.101 | 0.091-0.111 | 0.13 | 0.12-14 |  |
| Recovered, treatment | 0 | § | 0 | § |  |
| Recovered, no treatment | 0 | § | 0 | § |  |
|  |  |  |  |  |  |
| Psychiatrist visit (>30 <45 mins) | 108.12 | 97.3-118.93 | † | † | 2007 NSMHWB, [(10)](#_ENREF_37), [(11)](#_ENREF_67), (12, 13) |
| Depressed, in treatment | 0.747 | 0.672-0.822 | 0.37 | 0.33-0.41 |  |
| Depressed, no treatment | 0 | § | 0.36 | 0.33-0.41 |  |
| Recovered, treatment | 0 | § | 0 | § |  |
| Recovered, no treatment | 0 | § | 0 | § |  |
|  |  |  |  |  |  |
| Psychologist visit (>60 mins) | 181.54 | 163.38-199.69 | † | † | 2007 NSMHWB, [(10)](#_ENREF_37), [(11)](#_ENREF_67), (12, 13) |
| Depressed, in treatment | 8.66 | 7.79-9.53 | 3.34 | 3.01-3.67 |  |
| Depressed, no treatment | 0 | § | 0 | § |  |
| Recovered, treatment | 0 | § | 0 | § |  |
| Recovered, no treatment | 0 | § | 0 | § |  |
|  |  |  |  |  |  |
| Total Service Use | 11.33 | 10.2-12.46 | 4.89 | 4.40-5.38 |  |
| Depressed, in treatment | 10.11 | 9.09-11.12 | 4.39 | 3.95-4.83 |  |
| Depressed, no treatment | 0.101 | 0.091-0.111 | 0.51 | 0.46-0.56 |  |
| Recovered, treatment | 0 |  | 0 | § |  |
| Recovered, no treatment | 0 |  | 0 | § |  |
|  |  |  |  |  |  |
| Utilities – AqoL-4D Values ^‡^ |  |  |  |  |  |
| Depressed, treatment | 0.1180 | Beta (α 56.91, β 425.40)  95% CI: 0.089-0.1468 | 0.1108 | Beta (α 31.29, β 251.15)  95% CI: 0.0743-0.1473 | 2007 NSMHWB |
| Depressed, no treatment | 0.1230 | Beta (α 271.25, β 1933.73)  95% CI: 0.1093-0.1367 | 0.1219 | Beta (α 17.22, β 124.02)  95% CI: 0.068-0.1757 | 2007 NSMHWB |
| Recovered, treatment | 0.1221 | Beta (α 306.74, β 2204.20)  95% CI: 0.1094-0.1350 | 0.1102 | Beta (α 8.41, β 67.92)  95% CI: 0.0404-0.1800 | 2007 NSMHWB |
| Recovered, no treatment | 0.1499 | Beta (α 1471.36, β 8342.99)  95% CI: 0.1429-0.1570 | 0.1725 | Beta (α 529.83, β 2541.63)  95% CI: 0.1591-0.1858 | 2007 NSMHWB |
|  |  |  |  |  |  |
| Discount rate | 3% | 0-5 | † | † | [(2)](#_ENREF_17) |

* National Survey of National Survey of Wellbeing (2007)

†Denotes the same value for each decision option.

‡ Assessment of Quality of Life-4D

§ No data available for this parameter from the 2007 NSMHWB e.g.no service or antidepressant use reported.
