# Supplementary material for: Depression in Working Adults: Comparing the Costs and Health Outcomes of Working When Ill
Source: PLoS One. 2014 Sep 2;9(9):e105430. doi: 10.1371/journal.pone.0105430 (PMC4152191; doi:10.1371/journal.pone.0105430)
Supplement: Table S3 — Data inputs and assumptions in presenteeism model, where estimates differ from absenteeism model. (DOCX) [file pone.0105430.s003.docx]

| Variable/Parameter | White Collar |  | Blue Collar |  |  |
| --- | --- | --- | --- | --- | --- |
| Miscellaneous Probabilities | Mean | Distribution/Range | Mean | Distribution/Range | Source |
| 3-mo primary care physician visits | 0.007 | Beta (α 0.37, β 49.30) | 0.008 | Beta (α 0.53, β 62.11) | 2007 NSMHWB^*^ |
| 3-mo psychiatrist visits | 0.0015 | Beta (α 0.015, β 9.87) | § | § | 2007 NSMHWB |
| 3-mo psychologist visits | 0.0045 | Beta (α 0.17, β 36.39) | § | § | 2007 NSMHWB |
| 3-mo antidepressant use |  |  |  |  |  |
| Total | 0.239 | Beta (α 8.28, β 9.93) | 0.069 | Beta (α 11.55, β 62.75) | 2007 NSMHWB |
| Depressed, treatment | 0.431 | Beta (α 0.017, β 36.39) | 0.332 | Beta (α 5.38, β 10.83) | 2007 NSMHWB |
| Depressed, no treatment | § | § | § | § | 2007 NSMHWB |
| Recovered, treatment | 1.0 | Beta (α 429.57, β 3.56) | 0.208 | Beta (α 2.46, β 8.54) | 2007 NSMHWB |
| Recovered, no treatment | 0.087 | Beta (α 4.84, β 50.74) | 0.014 | Beta (α 0.97, β 52.75) | 2007 NSMHWB |
|  |  |  |  |  |  |
| Lost Productive Time |  |  |  |  |  |
| Depressed, treatment | 738.12 | 664.3-811.9 | 490.04 | 441.04-539.04 | [(7)](#_ENREF_2), [(8)](#_ENREF_22), [(9)](#_ENREF_21) |
| Depressed, no treatment | 938.28 | 844.5-1032.1 | 630.05 | 567.05-693.06 | [(7)](#_ENREF_2), [(8)](#_ENREF_22), [(9)](#_ENREF_21) |
| Recovered, treatment | 367.00 | 330.3-403.7 | 256.71 | 231.04-282.38 | [(7)](#_ENREF_2), [(8)](#_ENREF_22), [(9)](#_ENREF_21) |
| Recovered, no treatment | 475.40 | 427.9-522.9 | 332.52 | 299.27-365.77 | [(7)](#_ENREF_2), [(8)](#_ENREF_22), [(9)](#_ENREF_21) |
|  |  |  |  |  |  |
| Service Use |  |  |  |  |  |
| 3-mo Antidepressant Use | 18.17 | 10.36-19.99 | 5.24 | 4.72-5.76 | 2007 NSMHWB, [(10)](#_ENREF_37), [(11)](#_ENREF_67) |
| Depressed, treatment | 32.68 | 29.4-35.9 | 25.17 | 22.65-27.69 |  |
| Depressed, no treatment | § | § | § | § |  |
| Recovered, treatment | 75.78 | 62.20-83.36 | 15.80 | 14.22-17.38 |  |
| Recovered, no treatment | 6.67 | 6.0-7.3 | 1.06 | 0.95-1.17 |  |
|  |  |  |  |  |  |
| Primary care physician visit (>5 < 25 mins) |  |  |  |  | 2007 NSMHWB, [(10)](#_ENREF_37), [(11)](#_ENREF_67), (12, 13) |
| Depressed, in treatment | 0.247 | 0.222-0.272 | 0.47 | 0.42-52 |  |
| Depressed, no treatment | 0.039 | 0.035-0.043 | 0 | 0 |  |
| Recovered, treatment | 0 | § | 0 | § |  |
| Recovered, no treatment | 0 | § | 0 | § |  |
|  |  |  |  |  |  |
| Psychiatrist visit (>30 <45 mins) |  |  |  |  | 2007 NSMHWB, [(10)](#_ENREF_37), [(11)](#_ENREF_67), (12, 13) |
| Depressed, in treatment | 0.406 | 0.365-0.447 | 0 | § |  |
| Depressed, no treatment | 0 | § | 0 | § |  |
| Recovered, treatment | 0 | § | 0 | § |  |
| Recovered, no treatment | 0 | § | 0 | § |  |
|  |  |  |  |  |  |
| Psychologist visit (>60 mins) |  |  |  |  | 2007 NSMHWB, [(10)](#_ENREF_37), [(11)](#_ENREF_67), (12, 13) |
| Depressed, in treatment | 7.41 | 6.66-8.15 | 0 | § |  |
| Depressed, no treatment | 0 | § | 0 | § |  |
| Recovered, treatment | 0 | § | 0 | § |  |
| Recovered, no treatment | 0 | § | 0 | § |  |
|  |  |  |  |  |  |
| Total Service Use | 8.11 | 7.29-8.92 | 0.47 | 0.42-0.52 |  |
| Depressed, in treatment | 8.07 | 7.26-8.87 | 0.47 | 0.42-0.52 |  |
| Depressed, no treatment | 0.039 | 0.035-0.043 | 0 | § |  |
| Recovered, treatment | 0 | § | 0 | § |  |
| Recovered, no treatment | 0 | § | 0 | § |  |
|  |  |  |  |  |  |
| Utilities – AqoL-4D Values‡ |  |  |  |  |  |
| Depressed, treatment | 0.1412 | Beta (α 35.97, β 218.69)  95% CI: 0.098-0.1840 | 0.1565 | Beta (α 372.74, β 2008.96)  95% CI: 0.1420-0.1711 | 2007 NSMHWB |
| Depressed, no treatment | 0.1501 | Beta (α 94.39, β 534.44)  95% CI: 0.1222-0.1780 | 0.1378 | Beta (α 40.70, β 254.69)  95% CI: 0.098-0.1771 | 2007 NSMHWB |
| Recovered, treatment | 0.1656 | Beta (α 291.51, β 1468.79)  95% CI: 0.1482-0.1830 | 0.1558 | Beta (α 7.94, β 43.04)  95% CI: 0.0571-0.2544 | 2007 NSMHWB |
| Recovered, no treatment | 0.1835 | Beta (α 1413.0, β 6286.34)  95% CI: 0.1749-0.1922 | 0.1948 | Beta (α 840.15, β 3472.74)  95% CI: 0.1830-0.2066 | 2007 NSMHWB |

* National Survey of National Survey of Wellbeing (2007)

†Denotes the same value for each decision option.

‡ Assessment of Quality of Life-4D

§ No data available for this parameter from the 2007 NSMHWB e.g.no service or antidepressant use reported.
