# Supplementary material for: Depression in Working Adults: Comparing the Costs and Health Outcomes of Working When Ill
Source: PLoS One. 2014 Sep 2;9(9):e105430. doi: 10.1371/journal.pone.0105430 (PMC4152191; doi:10.1371/journal.pone.0105430)
Supplement: Table S4 — Absenteeism and presenteeism days and associated lost productive time by occupation type. (DOCX) [file pone.0105430.s004.docx]

|  |  | **Absenteeism** |  | **Presenteeism** |  |
| --- | --- | --- | --- | --- | --- |
|  | Daily Wage  (Hourly wage/Average daily Hours) | N days per quarter | Lost Productive Time  ($)  Daily Wage * N days per quarter | N days per  quarter | Lost Productive Time  ($)  Daily Wage * N days per quarter |
|  |  |  |  |  |  |
| **Blue Collar (N=210)** |  |  |  |  |  |
| Depressed, in treatment | $170.2 | 14.3 | 2426.20 | 2.86 | 486.40 |
| Depressed, not in treatment |  | 5.2 | 881.21 | 3.70 | 630.05 |
| Recovered, in treatment |  | 1.5 | 251.05 | 1.51 | 256.71 |
| Recovered, not in treatment |  | 1.8 | 306.36 | 1.95 | 332.52 |
|  |  |  |  |  |  |
|  |  |  |  |  |  |
| **White Collar (N=634)** |  |  |  |  |  |
| Depressed, in treatment | $215.2 | 17.4 | 3067.87 | 3.09 | 665.50 |
| Depressed, not in treatment |  | 9.3 | 1441.68 | 3.90 | 839.66 |
| Recovered, in treatment |  | 1.1 | 338.90 | 1.53 | 328.43 |
| Recovered, not in treatment |  | 1.3 | 416.17 | 1.98 | 425.43 |
